# Benefits of a sustained community-based psychiatric intervention on viral exposure among people who inject drugs: The ANRS Drive-Mind 2 cohort study in Hai Phong, Vietnam

**DOI:** 10.1371/journal.pmen.0000631

**Published:** 2026-07-08

**Authors:** Sao Mai Le, Philippe Trouiller, Giang Hoang Thi, Huong Duong Thi, Oanh Khuat Thi Hai, Khue Pham Minh, Roselyne Vallo, Delphine Rapoud, Catherine Quillet, Thuy Linh Nguyen, Quang Duc Nguyen, Tuyet Thanh NhamThi, Vinh Vu Hai, Jean-Pierre Moles, Didier Laureillard, Don C. Des Jarlais, Nicolas Nagot, Laurent Michel

**Affiliations:** 1 Hai Phong University of Medicine and Pharmacy, Hai Phong, Vietnam; 2 CESP Inserm UMRS, Paris Saclay University, Pierre Nicole Center, French Red Cross, Paris, France; 3 Supporting Community Development Initiatives, Hanoi, Vietnam; 4 Pathogenesis and control of chronic and emerging infections, University of Montpellier, Inserm, Etablissement Français du Sang, University of Antilles, Montpellier, France; 5 Dept of Infectious and tropical diseases, Viet Tiep Hospital, Hai Phong, Vietnam; 6 Infectious Diseases Department, Caremeau University Hospital, Nîmes, France; 7 New York University, College of Global Public Health, New York, New York, United States of America; PLOS: Public Library of Science, UNITED KINGDOM OF GREAT BRITAIN AND NORTHERN IRELAND

## Abstract

Community-based psychiatric interventions for people who inject drugs (PWID) have been proven to be feasible and efficient in low-middle income countries (LMIC) where psychiatric resources are scarce and stigma important. We aimed to show that, on top of mental health improvement, PWID initially diagnosed with psychiatric symptoms and who received a sustained community-based psychiatric intervention were comparable to a control population of PWID in terms of HIV/HCV exposure. In Hai Phong, Vietnam, PWID currently or previously diagnosed with psychiatric symptoms were invited to be recruited in a 12-month follow-up cohort and proposed a community-based psychiatric and harm reduction intervention supported by peers and were compared after intervention to control PWID free from any psychiatric diagnosis who benefited from harm reduction interventions. HIV/HCV exposure was assessed using a composite score taking into account sex and drug-related risk behaviors, weighted according to the viral exposure risk. Psychiatric symptoms, severity of drug use and quality of life were also assessed at each visit. After a 12-month follow-up between March 2022 and April 2023, viral exposure among PWID diagnosed with psychiatric symptoms was considered significantly non-inferior to that of the control group. Their mental health status was significantly improved and severity of drug use or quality of life did not differ significantly from controls. In LMIC, community-based psychiatric intervention supported by trained peers is feasible and efficient for most dimensions. It may represent a valuable alternative to the classical mental health system.

## Introduction

Co-occurrence of psychiatric disorders and substance use is common among people who use drugs (PWUD), particularly among those who use stimulants [1–4]. These PWUD suffer of reduced quality of life, increased HIV/HCV exposure but also poorer health-seeking behaviors and late access to methadone treatment for those opiate-dependent [5–9].

Developing a comprehensive approach from screening to care for psychiatric issues is now a major recommendation [10–12]. Nevertheless, due to scarcity of mental health settings and professionals in many parts of the world, access to psychiatric care is limited, particularly for key populations living in precarious social situations and facing important stigma [13–16]. The need for alternative interventions has been emphasized, including community-based interventions and peer-support, after skill transfer and task shifting [17–20].

In a previous survey conducted in the city of Hai Phong (Vietnam) in 2019, we could show that a community-based psychiatric intervention for PWID was feasible and efficient in the Vietnamese context (DRIVE-Mind cohort study) [21]. Psychiatric consultations and treatments were provided free of charge to the PWID who agreed to participate to the cohort. Peers were involved at each step of the project implementation and provided full support to the psychiatric follow-up. Most of the participants diagnosed with psychiatric symptomsaccepted to be enrolled in a cohort and nearly 80% of the PWID who completed the one-year follow-up were clinically improved (CGI scale). However, the impact on the drug and sex-related risk behaviors was not measured.

The objective of this second phase of the DRIVE-Mind project (DRIVE-Mind 2) was to show that PWID initially diagnosed with a major depressive disorder, a psychotic disorder and/or had a suicide risk and who received a sustained community-based psychiatric intervention are comparable to a population of PWID free of these disorders in terms of HIV/HCV exposure.

## Materials et methods

### Ethics Statement

The research protocol was approved by the Institutional Ethics Committees of the Hai Phong Medicine and Pharmacy Faculty in Vietnam. Individual written informed consent was obtained from all participants prior to participation in each Respondent Driving Sampling (RDS) survey and in the cohort study.

### The DRIVE Project (Drug use and infections in Vietnam)

Starting in 2016, a research program (DRIVE project) was implemented among PWID in Hai Phong, aiming to end the HIV epidemic in this population through a combined community-based intervention including repeated HIV testing, linkage to care (antiretroviral therapy, methadone, mental health), harm reduction and administrative support (detail in [22]). Criteria for inclusion in the different RDS surveys were: age 18 or over, self-reported current drug injection, conﬁrmed by presence of recent skin injection marks and positive urinalysis for heroin and/or methamphetamine, residence in Hai Phong, and ability to provide informed consent.

### The DRIVE-Mind project

#### DRIVE-Mind 1 study.

The objective of the DRIVE-Mind 1 study was to assess the impact of a psychiatric intervention at community level on the evolution of symptoms among PWID in Hai Phong and evaluate access to and retention in care for people with psychiatric conditions (detail in [21]). PWID participating in the larger DRIVE project were screened for psychiatric symptoms during a bi-annual follow-up visits in March 2019 and invited to be recruited in the Drive-Mind 1 cohort if a condition requiring a psychiatric intervention was diagnosed (detail in [21]). Among the 1212 participants screened, 271 (22%) were eligible for a psychiatric intervention and 233 (86%) were enrolled and signed an informed consent, 170 completed the 12 months follow-up (73%).

#### DRIVE-Mind 2 study.

**Recruitment procedure.** DRIVE-Mind 2 study participants were recruited from March 2022 to May 2022. PWID eligible for recruitment in the DRIVE-Mind 2 study were invited to a one-year follow-up, which included a visit at 6 months and a ﬁnal visit at the end of the project (12 months). Trained psychiatrists from Hai Phong school of Medicine and Pharmacy systematically administered the following modules of the Mini-International Neuropsychiatric Interview (MINI 5.0.0) [23]: major depressive disorder, psychotic disorder or suicide risk. These psychiatrists also provided their clinical input on the MINI diagnosis and assessed the mental health status of every PWID to detect any other disorder requiring a psychiatric intervention.

### Psychiatric cohort

All the participants of the Drive-Mind 1 survey were eligible for the enrollment in the psychiatric cohort, independently of their current psychiatric status at the inclusion visit (still symptomatic or clinically recovered). Were also eligible the candidates from the control group diagnosed at inclusion with psychiatric symptoms requiring support and treatment.

Participants enrolled in the control group and diagnosed at any step of the one-year follow-up with significant psychiatric symptoms would also be eligible for the psychiatric intervention.

### Control Group

PWID of the DRIVE cohort screened negative for any psychiatric disorder at the inclusion visit of the DRIVE-Mind 1 cohort were recalled and invited to be enrolled in the control group of the DRIVE-Mind 2 survey. They had to be free of any significant psychiatric syndrome requiring support and treatment.

Recruitment in the control group was carried out until 200 PWID living with HIV and 200 PWID not infected with HIV were enrolled, to compare HIV outcomes in the psychiatric cohort and control group in a second phase.

### Intervention for the psychiatric cohort and the control group

#### Psychiatric cohort.

PWID enrolled in the psychiatric cohort received the same intervention than during the DRIVE-Mind 1 study (see section “DRIVE-Mind 1 study” and [21]). The intervention took place in two different houses rented by community-based organizations (CBOs) with support of the non-government organization Supporting Community Development Initiatives (SCDI). The same two psychiatrists from the mental health department were involved in the assessment and follow-up (support, therapeutic education, prescription) of the PWID included in the cohort. The psychiatrists’ intervention took place in the CBO offices. The frequency of appointments with participants for clinical care follow-up was scheduled according to the clinical situation of each PWID and varied from once a week to once a month. Two antidepressants (sertraline and mirtazapin), four antipsychotics (risperidone, olanzapine, sulpiride, quetiapine), and melatonine for sleeping disorders were available and issued free of charge by the psychiatrists at the end of consultations. Due to very restrictive regulation in Viet Nam, benzodiazepines were not used. When needed, participants could be hospitalized in the mental health department and associated fees paid by research funds.

The same CBO and members in the DRIVE-Mind 1 study participated in DRIVE-Mind 2 study [21]. Their training on mental health intervention was detailed previously [21] and their tasks remained the same than during the DRIVE-Mind 1 study (Table A in S1 Appendix).

The salary for their intervention was 4,500,000 VND (179 US dollars) per month.

### Control group

The control group did not receive any psychiatric intervention. All PWID met again a psychiatrist at M6 and M12 follow-up visits for evaluation. If a psychiatric condition was detected at the M6 follow-up, the PWID was invited to join the psychiatric cohort.

### Psychiatric cohort and control group

The CBO members invited all PWID of the DRIVE-Mind 2 study to: linkage to HIV care and methadone maintenance treatment (MMT), administrative support (health insurance, identity card, resident card), harm reduction intervention (counselling, sterile needles-syringes, condoms).

### Data collection

Data were collected during face-to-face interviews with trained interviewers at enrollment visit (M0 visit), after 6 months (M6 visit) and after 12 months (M12 visit, end of study).

### Outcomes and methods of measurement

#### Primary endpoint.

**HIV/HCV exposure: the viral exposure score**. The viral exposure score is the sum of the answers to several HIV/HCV-related risk behaviors questions, weighed according to the significance of the risk (timeframe: last 6 months). Questions considered are: drug injection/ current methadone treatment (weight: 2), syringe/needle sharing (weight: 3); frontloading/backloading drug before injection (weight: 3); sharing solution for dissolution or syringe rinse (weight: 2); inconsistent condom use with at risk primary partner (partner living with HIV and not treated with ARV or HIV status unknown)(weight: 2); inconsistent condom use with casual partner (weight: 2); pipe sharing (weight:1), using or not equipment to stop bleeding after injection (weight:1) (Table B in S1 Appendix) and was measured at M0, M6 and M12 visits. Primary endpoint was based solely on score measured at M12 visit.

### Additional outcomes

Quality-of-life was measured at M0, M6 and M12 via the 5-level and 5 dimensions EuroQol instrument (EQ5D5L) which is a standardized measure of health status with 5 components (anxiety, pain/discomfort, mobility, self-care, usual activities), and a score for perceived health where the participant is asked to score current health on a scale from 0 (worst health imaginable) to 100 (best health imaginable).

Two versions of the clinical global improvement (CGI) scale were used: at baseline the CGI Severity scale (CGI-S) to assess the initial severity of the illness; at M6 and M12 visits the CGI Improvement scale (CGI-I) to evaluate the improvement in clinical situation compared to cohort initiation.

At each visit, data on drug use, sexual behaviors and access to drug and HIV-related services were collected in face-to-face structured interviews by trained CBO staff. Data on gender was collected via self-report.

HIV seroconversion was ascertained by administering HIV rapid tests. Detectable HIV viral load (VL) was ascertained when above 1000 cp/mL. HCV infection was ascertained by an HCV positive rapid tests for HCV-seronegative participants and HCV reinfection by a detectable VL at M12 for HCV-seropositive VL undetectable participants at baseline.

### Data analysis

To ensure that our analysis is as conservative as possible and highlight differences between groups in terms of viral exposure, we excluded from the main analysis all the participants from the control group who were newly diagnosed with a psychiatric disorder at M6 visit. All the participants who benefited from the intervention and completed the M12 visit were included in the analysis, whether or not they completed the M6 visit.

To compare the viral exposure scores we calculated the difference between the score among psychiatric cohort and the score among the control group at the end of follow-up and computed the 95% confidence interval (CI) of this difference. If the upper limit of the 95% CI was less than the acceptable difference of 0.4 points, the viral exposure score would be considered non-inferior in the control group (one sided testing). This score is theoretically ranging from 1 to 16, with a standard deviation estimated at 1.1, based on a preliminary study. Therefore, we estimated that a total 400 participants at M12 visit will provide at least 80% power (alpha risk = 0.05) and would allow for HIV sub-group analysis if needed. We performed a multiple linear regression including the viral exposure score as the explained variable and intervention group with potential confounders as the explanatory variables. Beta-coefficients and their 95% CI were computed to gauge the number of points in the viral exposure score associated with being in the psychiatric cohort. An acceptable difference for the beta-coefficients at M12 visit was also set to 0.4 point in order to conclude to a non-inferiority between the two groups (primary endpoint).

While not being a primary outcome, quality of life scale ratings was compared using the same method. The estimated standard deviation for this 100-pts range scale was 15-pts, and a 5-pts acceptable delta was set to conclude to a non-inferiority between the two groups.

## Results

**Fig 1:**
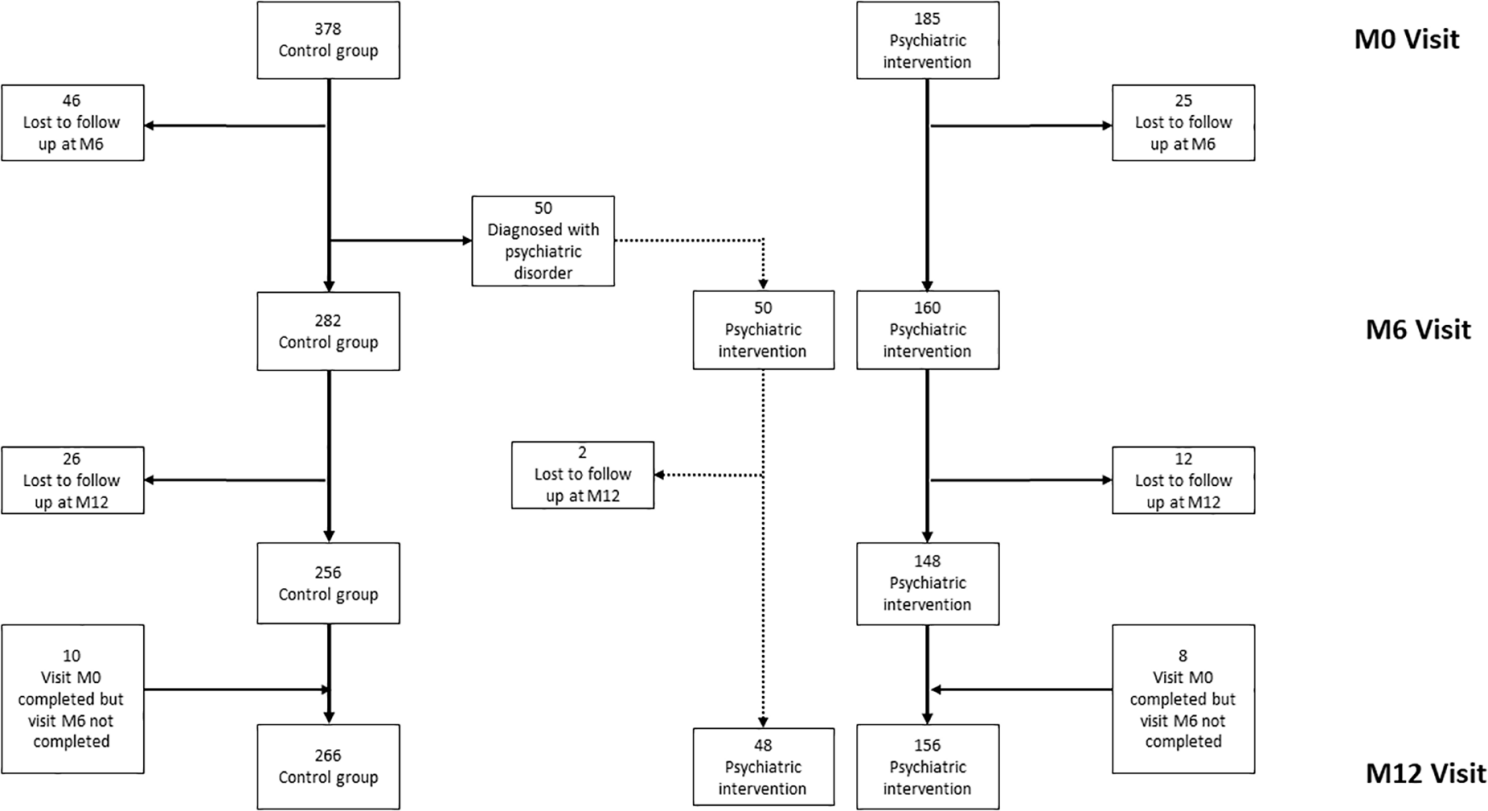
Flow chart. Between 20 March 2022 and 7 May 2022, 563 subjects were enrolled: 378 PWID in the control group (198 living with HIV), 185 in the psychiatric cohort (77 living with HIV).

During M6 visit, 50 PWID from the control group were diagnosed with a psychiatric condition requiring intervention and joined the psychiatric cohort. At M12 visit, 266 PWID from the control group came back and 204 from the psychiatric cohort (156 diagnosed at M0 visit and 48 newly diagnosed at M6 visit). Even though participants from the psychiatric cohort did not attend M6 visit for different reasons, they benefited from psychiatric support during the 12 months of the intervention. Therefore 156 participants from the psychiatric cohort were included in the analyses (Fig 1).

Participants from the psychiatric cohort were older than controls (mean age was 50 years), were less often married, and declared less often having a health insurance card or a regular salary (Table 1). Among them, 108 had participated in the DRIVE-Mind 1 study, and 48, who had no psychiatric disorders prior to DRIVE-Mind 2, had been invited to join the control group but were diagnosed with a psychiatric disorder at the initiation of the DM2 project and were therefore referred to the psychiatric cohort (their characteristics are presented in Table C in S1 Appendix).

**Table 1:** Socio-demographic characteristics of participants at baseline.

	Total (n = 422)	Control group (n = 266)	Psychiatric cohort (n = 156)	p
**Age (mean (SD))**	48.36 (7.70)	46.95 (6.82)	50.78 (8.51)	<0.001
**Gender: Male (%)**	393 (93.1)	252 (94.7)	141 (90.4)	0,132
**Marital status (%)**				0,001
single	110 (26.1)	70 (26.3)	40 (25.6)	
legally married	187 (44.3)	135 (50.8)	52 (33.3)	
living maritally	13 (3.1)	6 (2.3)	7 (4.5)	
separated	104 (24.6)	50 (18.8)	54 (34.6)	
widowed	8 (1.9)	5 (1.9)	3 (1.9)	
**Having a health insurance (%)**	307 (72.7)	207 (77.8)	100 (64.1)	0,003
**Arrested in the last 6 months (%)**	9 (2.1)	5 (1.9)	4 (2.6)	0,904
**Source of income**				
regular salary	113 (26.8)	84 (31.6)	29 (18.6)	0,005
temprorary work/self employement	193 (45.7)	124 (46.6)	69 (44.2)	0,709
support from family/relatives	185 (43.8)	105 (39.5)	80 (51.3)	0,024
No income	6 (1.4)	2 (0.8)	4 (2.6)	0,275

### Psychiatric symptoms in the psychiatric cohort (Table 2)

At cohort initiation, depression was the most frequent psychiatric diagnosis and was detected in 40% of the participants. Retention rate in the psychiatric cohort was high with 156/185 (84%) of them coming back at M12 visit. The frequency of all psychiatric diagnosis was significantly reduced between M0 and M12. Among those showing a psychiatric disorder, 132 (85%) were very much or much clinically improved at M12 visit (using CGI scale), 13 (8%) minimally improved, and 2 (1%) unchanged.

**Table 2:** Evolution of psychiatric disorders between M0 and M12.

	Psychiatric cohort (N = 156)		
	M0	M12	p**
Current major depressive episode (%)	62 (39.7)	19 (12.2)	<0.001
Current psychotic disorder (%)	38 (24.4)	18 (11.5)	0,002
Suicide risk (%)			<0.001
None	106 (67.9)	116 (74.4)	
Low	41 (26.3)	32 (20.5)	
Moderate	2 (1.3)	1 (0.6)	
High	7 (4.5)	7 (4.5)	
One disorder or more* (%)	76 (48.7)	31 (19.9)	<0.001

* among the following: current depressive episode, current psychotic episode, suicidal risk moderate or high

**McNemar test

### Viral exposure score (Table 3a and Table 3b)

**Fig 2:**
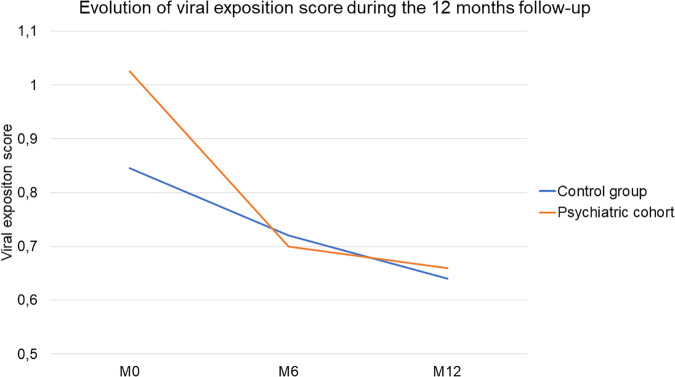
Viral exposition score.

Between M0 and M12 visits the viral exposure score decreased significantly for both groups (p > 0.001), and the score in the psychiatric cohort was significantly non-inferior to the one in the control group at M12, even though it was slightly higher (0.66 vs 0.64, means difference [IC 95%]: -0.02 [-0.16; 0.22]) (Table 3 and Fig 2). The difference in score between the two groups at M0 was mainly explained by the fact that PWID from the psychiatric cohort were more often injecting heroin while not treated with methadone, and that they were more often sharing pipe to smoke meth. The overall population showed very low levels of risky sexual behaviors (Table D in S1 Appendix).

**Table 3:** Bivariate analysis: comparison of viral exposure score between psychiatric cohort and control group. b. Multivariate analysis: factors associated with viral exposure score at M12 (including socio-demographic potential confounders).

	M0		M12		
	Control group (n = 266)	Psychiatric cohort (n = 156)	Control group (n = 250**)	Psychiatric cohort (n = 139**)	difference of means [CI 95%]*
**Viral exposure score** **(mean,sd)**	0.85 (1.03)	1.03 (1.21)	0.64 (0.93)	0.66 (0.94)	-0.02 [-0.21; 0.17]
	**Beta coefficient**		**95% IC**		*p*
**Being in the psychaitric cohort**	**-0,04**		**[-0.23; 0.15]**		*0,68*
*Age (for a 10 years increase)*	*0,1*		*[-0.02; 0.22]*		*0,11*
*Having a Regular salary*	*-0,07*		*[-0.27; 0.13]*		*0,45*
*Having an Insurance card*	*-0,19*		*[-0.60; 0.01]*		*0,06*

* IC95% computed by bootstrap basic method

** 33 participants with missing value for one or more item of the composite score.

Regarding the primary endpoint of this study, multivariate analysis confirmed the non-inferiority of the viral exposure score in the psychiatric cohort, when adjusting for age, regular salary, or having a health insurance card (Beta coefficient [IC 95%]: -0.04[-0.23; 0.15]) (Table 3b).

### Severity of substance use (Table 4)

Controls and PWID from the psychiatric cohort did not differ significantly in terms of severity of substance use at M12 visit. However, the use of heroin and methamphetamine seemed to decrease between M0 and M12 in both groups, but most notably in the psychiatric cohort for methamphetamine use in the last 6 months (14.6% at M12 vs 21.8% at M0, p = 0.031).

**Table 4:** Drug use at M0 and M12 visit.

	**M0**		**M12**		
	Control group	Psychiatric cohort	Control group	Psychiatric cohort	p**
	266	156	253*	144*	
**Heroin injection in the last 6 months (%)**	115 (43.2)	68 (43.6)	89 (35.2)	51 (35.4)	0,087
**Heroin injection in the last 30 days (number of days)**	10.00 [4.00, 30.00]	17.50 [3.75, 30.00]	10.00 [3.00, 30.00]	14.00 [3.50, 30.00]	0,235
**Heroin injection (last 30 days)**	114 (42.9)	68 (43.6)	89 (35.2)	51 (35.4)	0,058
**Meth use in the last 6 months (%)**	27 (10.2)	34 (21.8)	24 (9.5)	21 (14.6)	0,031
**Meth use (last 30 days)**	24 (9.0)	31 (19.9)	24 (9.5)	21 (14.6)	0,11
**Meth use ine the last 30 days (number of days, median [IQR])**	3.00 [1.00, 6.50]	2.00 [1.00, 4.75]	3.00 [2.00, 10.50]	2.00 [1.00, 4.00]	0,163
**Regular meth use (>3 times last 30 days)**	12 (4.5)	13 (8.3)	8 (3.2)	7 (4.9)	0,181
**Alcohol misuse (Audit-C)**	41 (15.4)	25 (16.0)	41 (16.2)	23 (16.0)	0,851
**Current MMT**	227 (85.7)	105 (67.3)	209 (82.6)	93 (64.6)	0,502

* loss of data (after data collection) on drug use for 25 participants at M12

** Mcnemar and paired t-tests comparing M0 and M12 data in the psychiatric cohort

meth: methamphetamine.

### HIV/HCV seroconversion, HIV viral load

HIV seropositivity rates were 51.5% in the control group (12.3% of them showed detectable VL at M0) and 42.3% in the psychiatric cohort (10.9% with detectable VL at M0). No participant seroconverted for HIV during the 12 months follow-up and rates of detectable VL were similar at M12 (9.1% in the control group, and 7.6% in the psychiatric cohort). HCV detectable VL rates were 10.4% at M12 in the control group (height new infections during the one year follow up) and 7.7% in the psychiatric cohort (four new infections).

### Quality of life (Table 5 and 5b)

Quality of life was rated significantly lower in the psychiatric cohort. Being in the psychiatric cohort was still associated with a 2.12 points decrease (IC95%: [-4.45; 0.07]) when adjusting for confounders. Since the value 5 is excluded from the 95% CI, we can conclude to a non-inferior quality of life rating in the psychiatric cohort. Regarding the 5 dimensions EuroQol instrument, scores of PWID from the psychiatric cohort were still significantly lower in every dimension except self-care at M12 visit, but differences between groups were seriously reduced (for detail, see Table E in S1 Appendix).

**Table 5:** Bivariate analysis: Quality of Life (N = 417 at M0 and N = 395 at M12). b. Multivariate analysis: factors associated with QoL scale at M12 (including socio-demographic potential cofounders, N = 394).

	M0			M12		
	Control group (N = 262)	Psychiatric cohort (N = 155)	difference of the means at M12 [IC 95%]*	Control group (253)**	Psychiatric cohort (N = 152)**	difference of the means at M12 [IC 95%]*
QoL Scale (mean,sd)	82,29	75,60	-6,67 [-9,09; -4,08]	81,65	77,38	-4.27 [-6.66; -2,05]
	**Beta coefficient**			**95% IC**	*p*	
**Being in the psychaitric cohort**	**-2,18**			**[-4.45; 0.07]**	*0,05*	
*Age*	*-0,26*			*[-0.40; -0.13]*	*<0.001*	
*Having a Regular salary*	*2,61*			*[0.27; 4.96]*	*0,02*	
*Having an Insurance card*	*0,22*			*[-2.12; 2.56]*	*0,85*	
*Current MMT*	*3,10*			*[0.55; 5.66]*	*0,01*	

* IC95% computed by bootstrap basic method

** 14 participants with missing value

## Discussion

The results of our study suggest that a sustained community-based psychiatric intervention is an efficient strategy to reach and treat PWID suffering from psychiatric disorders, a highly stigmatized population. It confirms the results of a preliminary study implemented in the same city in 2019–2020, with a good adherence to the intervention (more than half of participants of the previous Drive-Mind study agreeing to come back to join the cohort 2 years later), a high retention rate (84%), and 90% of the PWID suffering from a psychiatric condition baseline being significantly clinically improved at the end of the study.

Compared to a control group of PWID free of any psychiatric symptom, PWID diagnosed with a psychiatric disorder were not significantly different in terms of viral exposure and HIV/HCV infection after the 12-month community-based psychiatric intervention.

Our study suggests that, in addition to a benefit on mental health, this sustained community-based psychiatric intervention is also associated with reduced drug-related risks and substance use (particularly a reduction in methamphetamine use), and improved quality of life in this population. Integrated care that includes co-located mental health care and drug/alcohol specialist treatment is positively correlated with improved treatment engagement and remission or abstinence from substance use in study participants with dual disorders [24,25]. Nevertheless, due to the cost of these integrated structures and scarcity of resources in most LMIC, alternative strategies have been suggested, including community-based interventions relying on peers or lay workers [11,12,26]. The DRIVE-Mind 1 study did show an improvement in mental health status but a very limited reduction in drug use (except for alcohol use) for the PWID recruited in the cohort over the 12-month follow-up period; and quality of life was only partly improved. Protracted periods of care, longer than 12 months, could be necessary to maximize the positive impact of the intervention on reducing drug-related risks [27] and integrated care is likely to reduce cost and improve outcome probably only for interventions lasting more than 12 months [28]. In our study population, most of the PWID (70%) recruited in the psychiatric cohort were engaged in psychiatric care since March 2019 with proposed consultations in the community with the same psychiatrist between Drive-Mind 1 et Drive-Mind 2 studies. Nevertheless, considering the study population joining the psychiatric cohort at Drive-Mind 2 cohort initiation and free of any previous psychiatric care, they were comparable to control group in terms of drug/alcohol use and quality of life at the end of the study. A possible explanation is that the COVID crisis have interfered with the impact of the psychiatric intervention in our Drive-Mind 1 study. Regarding quality of life, if it did not significantly differ from controls for the global self-rating of quality of life, but were still different across dimensions of the EuroQol instrument. Participants of the psychiatric cohort were older, were very less likely to have a salary and were less often treated with methadone than controls, which may partly explain these results. Access to MMT treatment was constantly lower among PWID of the psychiatric cohort, which is a major concern and need further investigation in our population sample. Suffering from a psychiatric disorder has already been identified in Vietnam as a factor associated with lower adherence and retention to MMT but we could expect an improved access to MMT at the end of the study [29].

The community-based psychiatric intervention we developed in this study rely on a constant and structured peer support. The majority of CBO members were involved for years, with regular training and supervision, but received salary depending on grants only. Their income is then subject to cessation or drastic decrease when projects are ending and they need to always anticipate with alternative or complementary jobs. Establishing a network of peers and ensuring official accreditation so that they can effectively deliver mental health services is critical, especially in LMIC where psychiatric resources are most often scarce, given the considerable support then can provide [30,31].

A limitation of this study is that our intervention was implemented in a relatively homogeneous population, in a single place, where a research hub has been developed for years. Therefore, the level viral exposure related to drug use and sexual practices in this population was even lower than expected among the newly diagnosed participants in the psychiatric cohort. Similar projects should be replicated in different places and populations.

## Conclusion

Our study shows that a sustained community-based psychiatric intervention is efficient not only for improving mental health status but also to reduce the HIV/HCV exposure and diminished quality of life usually associated with psychiatric disorders. This model of care is then convincing but still faces barriers related to the availability of mental health professionals and MMT. Micro-costing analyses might further convince health authorities to implement this community-based intervention and to make peer workers status official.

## Supporting information

S1 Appendix Appendix. (DOCX)

S1 Checklist Inclusivity in global research questionnaire. (DOCX)

## Abbreviations

ANRS: French Agency for Research on AIDS and Viral Hepatitis
CBO: Community-based organization
CGI: Clinical global impression scale
DRIVE: Drug-Related Infections in ViEtnam
EQ5D5L: 5 levels/5 dimensions EuroQol instrument
HCV: Hepatitis C virus
HIV: Human immunodeficiency virus
LMICs: Low-middle income countries
MINI: MINI international neuropsychiatric interview
MMT: Methadone maintenance treatment
NIDA: National Institute on Drug Abuse
PWID: People who inject drugs
PWUD: People who use drugs
RDS: Respondent driven sampling
SCDI: Supporting community development initiatives
VL: Viral load
VND: Vietnamese dong

## References

[1] Schuckit MA. Comorbidity between substance use disorders and psychiatric conditions. Addiction. sept 2006;101(Suppl 1):76–88. doi: 10.1111/j.1360-0443.2006.01592.x 16930163

[2] Paulus MP, Stewart JL. Neurobiology, Clinical Presentation, and Treatment of Methamphetamine Use Disorder: A Review. JAMA Psychiatry. 2020;77(9):959–66. doi: 10.1001/jamapsychiatry.2020.0246 32267484 PMC8098650

[3] McKetin R, Leung J, Stockings E, Huo Y, Foulds J, Lappin JM, et al. Mental health outcomes associated with of the use of amphetamines: A systematic review and meta-analysis. EClinicalMedicine. 2019;16:81–97. doi: 10.1016/j.eclinm.2019.09.014 31832623 PMC6890973

[4] Grant BF, Saha TD, Ruan WJ, Goldstein RB, Chou SP, Jung J. Epidemiology of DSM-5 Drug Use Disorder: Results From the National Epidemiologic Survey on Alcohol and Related Conditions–III. JAMA Psychiatry. 2016;73(1):39. doi: 10.1001/jamapsychiatry.2015.2132 26580136 PMC5062605

[5] Iskandar S, Kamal R, De Jong CA. Psychiatric comorbidity in injecting drug users in Asia and Africa. Curr Opin Psychiatry. 2012;25(3):213–8. doi: 10.1097/YCO.0b013e3283523d66 22449767

[6] Remien RH, Stirratt MJ, Nguyen N, Robbins RN, Pala AN, Mellins CA. Mental health and HIV/AIDS: the need for an integrated response. AIDS. 2019. doi: 10.1097/QAD.0000000000002227 PMC6635049 30950883

[7] Levintow SN, Pence BW, Powers KA, Sripaipan T, Ha TV, Chu VA, et al. Estimating the Effect of Depression on HIV Transmission Risk Behaviors Among People Who Inject Drugs in Vietnam: A Causal Approach. AIDS Behav. 2021;25(2):438–46. doi: 10.1007/s10461-020-03007-9 32833193 PMC7444452

[8] Levintow SN, Pence BW, Powers KA, Breskin A, Sripaipan T, Ha TV, et al. Depression, antiretroviral therapy initiation, and HIV viral suppression among people who inject drugs in Vietnam. J Affect Disord. 2021;281:208–15. doi: 10.1016/j.jad.2020.12.024 33333474 PMC7855445

[9] Yen Y-F, Lai H-H, Kuo Y-C, Chan S-Y, Chen L-Y, Chen C-C, et al. Association of depression and antidepressant therapy with antiretroviral therapy adherence and health-related quality of life in men who have sex with men. PLoS One. 2022;17(2):e0264503. doi: 10.1371/journal.pone.0264503 35213633 PMC8880848

[10] Delany-Moretlwe S, Cowan FM, Busza J, Bolton-Moore C, Kelley K, Fairlie L. Providing comprehensive health services for young key populations: needs, barriers and gaps. J Int AIDS Soc. 2015;18(2 Suppl 1):19833. doi: 10.7448/IAS.18.2.19833 25724511 PMC4344539

[11] Patel V, Chisholm D, Parikh R, Charlson FJ, Degenhardt L, Dua T, et al. Addressing the burden of mental, neurological, and substance use disorders: key messages from Disease Control Priorities, 3rd edition. Lancet. 2016;387(10028):1672–85. doi: 10.1016/S0140-6736(15)00390-6 26454360

[12] Saxena S, Thornicroft G, Knapp M, Whiteford H. Resources for mental health: scarcity, inequity, and inefficiency. Lancet. 2007;370(9590):878–89. doi: 10.1016/S0140-6736(07)61239-2 17804062

[13] Li L, Lin C, Feng N, Nguyen DB, Cao W, Le AT, et al. Stigma Related to HIV and Drug Use: Layers, Types, and Relations to Mental Health. AIDS Behav. 2020;24(8):2347–54. doi: 10.1007/s10461-020-02794-5 31970581 PMC7374055

[14] Lan C, Lin C, Thanh DC, Li L. Drug‐related stigma and access to care among people who inject drugs in Vietnam. Drug and Alcohol Review. 2018;37(3):333–9. doi: 10.1111/dar.12589 28762584 PMC5794669

[15] Tran HV, Filipowicz TR, Landrum KR, Nong HTT, Tran TTT, Pence BW, et al. Stigma experienced by people living with HIV who are on methadone maintenance treatment and have symptoms of common mental disorders in Hanoi, Vietnam: a qualitative study. AIDS Res Ther. 2022;19(1):63. doi: 10.1186/s12981-022-00491-y 36517849 PMC9753276

[16] Hammarlund R, Crapanzano KA, Luce L, Mulligan L, Ward KM. Review of the effects of self-stigma and perceived social stigma on the treatment-seeking decisions of individuals with drug- and alcohol-use disorders. Subst Abuse Rehabil. 2018;9:115–36. doi: 10.2147/SAR.S183256 30538599 PMC6260179

[17] Satinsky EN, Kleinman MB, Tralka HM, Jack HE, Myers B, Magidson JF. Peer-delivered services for substance use in low- and middle-income countries: A systematic review. Int J Drug Policy. 2021;95:103252. doi: 10.1016/j.drugpo.2021.103252 33892281

[18] Kang KI, Kang CM. Roles and Effects of Peer Recovery Coach Intervention in the Field of Substance Abuse: An Integrative Literature Review. Asian Nurs Res (Korean Soc Nurs Sci). 2022;16(5):256–64. doi: 10.1016/j.anr.2022.10.001 36243312

[19] Chang J, Shelly S, Busz M, Stoicescu C, Iryawan AR, Madybaeva D, et al. Peer driven or driven peers? A rapid review of peer involvement of people who use drugs in HIV and harm reduction services in low- and middle-income countries. Harm Reduct J. 2021;18(1):15. doi: 10.1186/s12954-021-00461-z 33536033 PMC7857348

[20] WHO. Comprehensive mental health action plan 2013-2030. Geneva: World Health Organization. 2021.

[21] Michel L, Le SM, Thi GH, Trouiller P, Thi HD, Thi Hai OK, et al. Assessment of a psychiatric intervention at community level for people who inject drugs in a low-middle income country: the DRIVE-Mind cohort study in Hai Phong, Viet Nam. Lancet Reg Health West Pac. 2021;18:100337. doi: 10.1016/j.lanwpc.2021.100337 35024661 PMC8669310

[22] Des Jarlais DC, Huong DT, Oanh KTH, Feelemyer JP, Arasteh K, Khue PM, et al. Ending an HIV epidemic among persons who inject drugs in a middle-income country: extremely low HIV incidence among persons who inject drugs in Hai Phong, Viet Nam. AIDS. 2020;34(15):2305–11. doi: 10.1097/QAD.0000000000002712 33048884 PMC8608372

[23] Sheehan DV, Lecrubier Y, Sheehan KH, Amorim P, Janavs J, Weiller E, et al. The Mini-International Neuropsychiatric Interview (M.I.N.I.): the development and validation of a structured diagnostic psychiatric interview for DSM-IV and ICD-10. J Clin Psychiatry. 59(Suppl 20):22–33. 9881538

[24] Glover-Wright C, Coupe K, Campbell AC, Keen C, Lawrence P, Kinner SA, et al. Health outcomes and service use patterns associated with co-located outpatient mental health care and alcohol and other drug specialist treatment: A systematic review. Drug Alcohol Rev. 2023;42(5):1195–219. doi: 10.1111/dar.13651 37015828 PMC10946517

[25] Schaefer JA, Cronkite RC, Hu KU. Differential relationships between continuity of care practices, engagement in continuing care, and abstinence among subgroups of patients with substance use and psychiatric disorders. J Stud Alcohol Drugs. 2011;72(4):611–21. doi: 10.15288/jsad.2011.72.611 21683043

[26] Bruckner TA, Scheffler RM, Shen G, Yoon J, Chisholm D, Morris J, et al. The mental health workforce gap in low- and middle-income countries: a needs-based approach. Bull World Health Organ. 2011;89(3):184–94. doi: 10.2471/BLT.10.082784 21379414 PMC3044251

[27] Proctor SL, Herschman PL. The continuing care model of substance use treatment: what works, and when is “enough,” “enough?”. Psychiatry Journal. 2014;2014:1–16. doi: 10.1155/2014/692423 PMC4007701 24839597

[28] Rocks S, Berntson D, Gil-Salmerón A, Kadu M, Ehrenberg N, Stein V, et al. Cost and effects of integrated care: a systematic literature review and meta-analysis. Eur J Health Econ. 2020;21(8):1211–21. doi: 10.1007/s10198-020-01217-5 32632820 PMC7561551

[29] Nong T, Hodgkin D, Trang NT, Shoptaw SJ, Li MJ, Hai Van HT, et al. A review of factors associated with methadone maintenance treatment adherence and retention in Vietnam. Drug Alcohol Depend. 2023;243:109699. doi: 10.1016/j.drugalcdep.2022.109699 36603363 PMC9851667

[30] Castedo de Martell S, Wilkerson JM, Howell J, Brown HS 3rd, Ranjit N, Holleran Steiker L, et al. The peer to career pipeline: An observational study of peer worker trainee characteristics and training completion likelihood. J Subst Use Addict Treat. 2024;159:209287. doi: 10.1016/j.josat.2023.209287 38160878 PMC10947928

[31] Cooper RE, Saunders KRK, Greenburgh A, Shah P, Appleton R, Machin K, et al. The effectiveness, implementation, and experiences of peer support approaches for mental health: a systematic umbrella review. BMC Med. 2024;22(1):72. doi: 10.1186/s12916-024-03260-y 38418998 PMC10902990

