# Supplementary material for: Benefits of a sustained community-based psychiatric intervention on viral exposure among people who inject drugs: The ANRS Drive-Mind 2 cohort study in Hai Phong, Vietnam
Source: PLOS Ment Health. 2026 Jul 8;3(7):e0000631. doi: 10.1371/journal.pmen.0000631 (PMC13345469; doi:10.1371/journal.pmen.0000631)
Supplement: S1 Appendix — (DOCX) [file pmen.0000631.s001.docx]

**S1 Appendix**

**Table A: CBO and psychiatrist tasks during the intervention**

| CBO tasks related to mental health | - Individual and group information, education and communication on mental health, mental disorders, their treatments, side effects of the treatments and time to action, adherence to treatment - Distribution of flyers on harm reduction for methamphetamine users (including psychiatric consequences) and on mental health for peers and their family - Recall appointments with psychiatrists and payment for transportation fees - Information to psychiatrists in case of unusual events or worrying situation - Offer of closer follow-up for subjects signaled by the psychiatrist and when possible contact the family - Collection of information about participants lost of follow-up or in case of poor adherence - Referral of severe cases to hospital and payment for hospitalization fees when necessary - Meetings with family to inform, support and educate when necessary - Contact between family and doctors. |
| --- | --- |
| Other CBO tasks | - Linkage to HIV care and MMT - Administrative support (health insurance, identity card, resident card) - Harm reduction intervention (counselling, clean needles-syringes, condoms) - Collection of data on drug use, sexual behaviors and use of drug-related facilities during face-to-face structured interviews |
| Psychiatrists from the mental health department | - Free psychiatric consultations on CBO site - Free prescription on CBO site - Free delivery of treatment by psychiatrists on CBO site - Coordination of the follow-up |

**Table B: Variables included in the viral exposure score and associated scoring value**

| **Variables included in the viral exposure score and associated scoring value** | | |
| --- | --- | --- |
| **Methadone and injection** | methadone and no injection | 0 |
|  | methadone and injection | 1 |
|  | no methadone and injection | 2 |
| **Syringe/needle sharing** | no | 0 |
|  | yes | 3 |
| **Frontloading/backloading drug before injection** | no | 0 |
|  | yes | 3 |
| **Water sharing (preparation, rinse)** | no | 0 |
|  | yes | 2 |
| **Use equipment to stop bleeding after injection** | yes | 0 |
|  | no | 1 |
| **Sharing pipe to smoke meth** | no | 0 |
|  | yes | 1 |
| **Inconsistent condom use with at risk primary partner** | no | 0 |
|  | yes | 2 |
| **Inconsistent condom use with casual partner** | no | 0 |
|  | yes | 2 |
|  |  | **max = 16** |
|  |  |  |

**Table C. Comparison of the characteristics of the control group with characteristics of the participants of the psychiatric cohort initially invited to join the control group but diagnosed with a psychiatric disorder**

| \|  \| Overall n=314 \| Control Group n=266 \| Controls included in the psychiatric cohort at M0 n=48 \| p \| \| --- \| --- \| --- \| --- \| --- \| \| **Heroin injection in the last 6 months (%)** \| 132 (42.0) \| 115 (43.2) \| 17 (35.4) \| 0,395 \| \| **Heroin injection in the last 30 days (number of days, median [IQR])** \| 10.00 [4.00, 30.00] \| 12.50 [4.00, 30.00] \| 10.00 [3.00, 30.00] \| 0,385 \| \| **Heroin injection (last 30 days)** \| 131 (41.7) \| 114 (42.9) \| 17 (35.4) \| 0,422 \| \| **Meth use in the last 6 months (%)** \| 33 (10.5) \| 27 (10.2) \| 6 (12.5) \| 0,816 \| \| **Meth use (last 30 days)** \| 30 ( 9.6) \| 24 ( 9.0) \| 6 (12.5) \| 0,626 \| \| **Meth use ine the last 30 days (number of days, median [IQR])** \| 3.50 [2.00, 7.00] \| 3.50 [2.00, 7.75] \| 4.00 [2.00, 6.75] \| 0,895 \| \| **Regular meth use (>3 times last 30 days)** \| 15 ( 4.8) \| 12 ( 4.5) \| 3 ( 6.2) \| 0,879 \| \| **Alcohol misuse (Audit-C)** \| 46 (14.6) \| 41 (15.4) \| 5 (10.4) \| 0,497 \| \| **Current methadone treatment** \| 261 (83.4) \| 227 (85.7) \| 34 (70.8) \| **0,02** \| \| **QoL (mean, SD)** \| 81.2 (10.6) \| 82.3 (9.5) \| 75.2 (13.8) \| **0.012** \| \| **Heroin injection in the last 6 months (%)** \| 101 (34.1) \| 89 (35.2) \| 12 (27.9) \| 0,45 \| \| **Heroin injection in the last 30 days (number of days, median [IQR])** \| 10.00 [3.00, 30.00] \| 10.00 [3.00, 30.00] \| 5.00 [1.75, 18.75] \| 0,246 \| \| **Heroin injection (last 30 days)** \| 101 (34.1) \| 89 (35.2) \| 12 (27.9) \| 0,45 \| \| **Meth use in the last 6 months (%)** \| 28 ( 9.5) \| 24 ( 9.5) \| 4 ( 9.3) \| 1 \| \| **Meth use (last 30 days)** \| 28 ( 9.5) \| 24 ( 9.5) \| 4 ( 9.3) \| 1 \| \| **Meth use ine the last 30 days (number of days, median [IQR])** \| 2.50 [1.75, 10.00] \| 3.00 [2.00, 10.50] \| 1.50 [1.00, 2.75] \| 0,216 \| \| **Regular meth use (>3 times last 30 days)** \| 9 ( 3.0) \| 8 ( 3.2) \| 1 ( 2.3) \| 1 \| \| **Alcohol misuse (Audit-C)** \| 47 (15.9) \| 41 (16.2) \| 6 (14.0) \| 0,882 \| \| **Current methadone treatment** \| 240 (81.1) \| 209 (82.6) \| 31 (72.1) \| 0,156 \| \| **QoL (mean, SD)** \| 81.2(10.2) \| 81.6 (10.1 \| 79.3 (10.7) \| 0.230 \| |
| --- | --- | --- | --- | --- | --- | --- | --- | --- | --- | --- | --- | --- | --- | --- | --- | --- | --- | --- | --- | --- | --- | --- | --- | --- | --- | --- | --- | --- | --- | --- | --- | --- | --- | --- | --- | --- | --- | --- | --- | --- | --- | --- | --- | --- | --- | --- | --- | --- | --- | --- | --- | --- | --- | --- | --- | --- | --- | --- | --- | --- | --- | --- | --- | --- | --- | --- | --- | --- | --- | --- | --- | --- | --- | --- | --- | --- | --- | --- | --- | --- | --- | --- | --- | --- | --- | --- | --- | --- | --- | --- | --- | --- | --- | --- | --- | --- | --- | --- | --- | --- | --- | --- | --- | --- | --- |

| **Table D. Bivariate analysis : comparison of viral exposure score between psychiatric cohort and control group** | | | | | | |
| --- | --- | --- | --- | --- | --- | --- |
|  | **M0** | |  | **M12** | |  |
|  | **Control group (n=266)** | **Psychiatric cohort (n=156)** | p* | **Control group (n=266)** | **Psychiatric cohort (n=156)** | p* |
| **Viral exposition score** |  |  |  |  |  |  |
| **Methadone and injection** |  |  | 0,016 |  |  | 0,028 |
| no injection | 151 (56.8) | 88 (56.4) |  | 164 (61.7) | 93 (59.6) |  |
| methadone and injection | 99 (37.2) | 46 (29.5) |  | 77 (28.9) | 34 (21.8) |  |
| no methadone and injection | 15 ( 5.6) | 22 (14.1) |  | 12 ( 4.5) | 17 (10.9) |  |
| NA | 1 ( 0.4) | 0 ( 0.0) |  | 13 ( 4.9) | 12 ( 7.7) |  |
| **Front/backloading drug before injection** |  |  |  |  |  | 0,226 |
| 0 | 265 (99.6) | 156 (100) |  | 253 (95.1) | 153 (91.7) |  |
| 1 | 1 ( 0.4) | 0 ( 0.0) | 1 | 0 ( 0.0) | 0 ( 0.0) |  |
| NA | 0 ( 0.0) | 0 ( 0.0) |  | 13 ( 4.9) | 13 ( 8.3) |  |
| **Syringe/needle sharing** |  |  | 0,787 |  |  | 0,275 |
| 0 | 266 (100) | 155 (99.4) |  | 252 (94.7) | 143 (91.7) |  |
| 3 | 0 ( 0.0) | 1 ( 0.6) |  | 1 ( 0.4) | 0 ( 0.0) |  |
| NA |  |  |  | 13 ( 4.9) | 13 ( 8.3) |  |
| **Water sharing (preparation, rinse)** | 0 ( 0.0) | 0 ( 0.0) | 1 | 0 ( 0.0) | 0 ( 0.0) | 1 |
| **Use equipment to stop bleeding after injection** |  |  | 0,966 |  |  | 0,094 |
| 0 | 180 (67.7) | 104 (66.7) |  | 199 (74.8) | 120 (76.9) |  |
| 1 | 84 (31.6) | 51 (32.7) |  | 54 (20.3) | 22 (14.1) |  |
| NA | 2 ( 0.8) | 1 ( 0.6) |  | 13 ( 4.9) | 14 ( 9.0) |  |
| **Sharing pipe to smoke meth** |  |  | 0,007 |  |  | 0,075 |
| 0 | 261 (98.1) | 144 (92.3) |  | 251 (94.4) | 139 (89.1) |  |
| 1 | 5 ( 1.9) | 12 ( 7.7) |  | 2 ( 0.8) | 5 ( 3.2) |  |
| NA | 0 ( 0.0) | 0 ( 0.0) |  | 13 ( 4.9) | 12 ( 7.7) |  |
| **Unconsistent condom use with at risk primary partner^1^** |  |  | 0,425 |  |  | 0,309 |
| 0 | 261 (98.1) | 152 (97.4) |  | 249 (93.6) | 240 (90.4) |  |
| 2 | 0 ( 0.0) | 1 ( 0.6) |  | 0 ( 0.0) | 0 ( 0.0) |  |
| NA | 5 ( 1.9) | 3 ( 1.9) |  | 17 ( 6.4) | 15 ( 9.6) |  |
| **Unconsistent condom use with casual partner** |  |  | 1 |  |  | 0,106 |
| 0 | 264 (99.2) | 155 (99.4) |  | 252 (94.7) | 142 (91.0) |  |
| 2 | 2 ( 0.8) | 1 ( 0.6) |  | 0 ( 0.0) | 2 ( 1.3) |  |
| NA | 0 ( 0.0) | 0 ( 0.0) |  | 14 ( 5.3) | 12 ( 7.7) |  |
|  |  |  |  |  |  |  |
|  |  |  |  |  |  |  |
| **Viral exposure score  (mean ,sd)** | 0.85 (1.03) | 1.03 (1.21) | 0.18 [-0.06 ; 0.41] | 0.6 (0.91) | 0.63 (0.96) | 0.03 [-0.16 ; 0.22] |
|  |  |  |  |  |  |  |
| *Chi-squared test for all categorical variables and difference of the means [IC 95 %] for viral exposure score | | | | |  |  |
| ^1^ unconsistent condom use with the primary partner reported as HIV+ and untreated or with unknown HIV status | | | | |  |  |

**Table E. Biavariate analysis of the 5 dimensions EuroQol instrument (EQ5D5L)**

|  |  |  |  |  |  |  |  |
| --- | --- | --- | --- | --- | --- | --- | --- |
|  | **M0** | |  |  | **M12** | |  |
|  | **Control group** | **Psychiatric cohort** | **p**** |  | **Control group** | **Psychiatric cohort** | **p** |
| **N*** | 264 | 156 |  |  | 253 | 144 |  |
| **Mobility (mean (SD))** | 0.02 (0.12) | 0.17 (0.43) | <0.001 |  | 0.01 (0.11) | 0.08 (0.29) | 0,002 |
| **Self-care (mean (SD))** | 0.00 (0.06) | 0.03 (0.18) | 0,018 |  | 0.01 (0.11) | 0.04 (0.23) | 0,084 |
| **Usual activities (mean (SD))** | 0.01 (0.09) | 0.06 (0.31) | 0,013 |  | 0.01 (0.09) | 0.05 (0.25) | 0,018 |
| **Pain (mean (SD))** | 0.04 (0.20) | 0.25 (0.49) | <0.001 |  | 0.09 (0.28) | 0.19 (0.46) | 0,007 |
| **Aniety (mean (SD))** | 0.02 (0.16) | 0.24 (0.53) | <0.001 |  | 0.02 (0.14) | 0.14 (0.42) | <0.001 |
|  |  |  |  |  |  |  |  |
| * loss of data on QoL after data collection for 2 participants at M0 and 25 participants at M12 | | | |  |  |  |  |
| ** test comparing the control group and the psychiatric cohort | | |  |  |  |  |  |
