# Supplementary material for: Benefits of a sustained community-based psychiatric intervention on viral exposure among people who inject drugs: The ANRS Drive-Mind 2 cohort study in Hai Phong, Vietnam
Source: PLOS Ment Health. 2026 Jul 8;3(7):e0000631. doi: 10.1371/journal.pmen.0000631 (PMC13345469; doi:10.1371/journal.pmen.0000631)
Supplement: Attachment — Submitted filename: Response to reviewers.docx [file pmen.0000631.s004.docx]

**Responses to reviewer 1**

**Reviewer #1:** Congratulations on the DRIVE-MIND2 project and an interesting article presenting the findings. That said, there are a few concerns for your consideration:

1- Language: In many instances, the authors use highly clinical language, but in others, the language is very 'community' - I would strongly suggest harmonizing language and terminology so that the article is consistent throughout. For example, the authors use "mental health problem" "disorder" and "condition". "mental health problem" (line 31) and "disorder" (appearing 47 times in the article) are stigmatizing and should be avoided. in most instances, the authors could simply say that psychiatric symptoms were detected.

**Thanks for this comment. We have followed the reviewer's recommendations as often as possible throughout the manuscript. In some cases, and because psychiatric symptoms were identified by certain modules of the MINI but not all, we can state that participants did not present with certain disorders, but not that they were free of all psychiatric disorders.**

In lines 239-240, the authors say that "all psychiatric disorders significantly decreased" - but a disorder can't be decreased; SYMPTOMS may abate, manifest less frequently, be undetectable or less detectable, etc, but the disorder itself can't be "decreased" in English.

**The sentence has been rewritten accordingly.**

Likewise, suggest avoiding "patients" (lines 132, 292) in favor of "study participants" or "PWID".

**The corrections have been made accordingly.**

There are many acronyms that should be spelled out: RDS (line 82), CBO (134), SCDI (135), MMT (155; the authors use methadone, methadone services, etc - use consistent terminology throughout the article), VL (191 - and viral load appears several times out long - either use the acronym consistently or spell out long every time). Some of these acronyms do not appear in he acronyms list a the end.

**The corrections have been made accordingly.**

line 144 has "CBO organizations" which means "community-based organizations organizations" - delete the "organizations" after CBO

**The correction has been made accordingly.**

When referring to study timelines, please use "12-month follow-up" (not 12 months follow up). (lines 35, 41, 287)

**The corrections have been made accordingly.**

Line 36 "intervention" not "interventions" (a 12-month ... intervention)

**The correction has been made accordingly.**

Line 61 "situations" not "situation" (living in precarious social situations)

**The correction has been made accordingly.**

Study participants can't be "proposed to be recruited" (lines 35, 100, 108, 124, 152, 154) - they were "INVITED to be recruited..."

**The corrections have been made accordingly.**

line 98-99 "PWID PARTICIPATING IN the larger..." not "PWID of the larger..."

**The correction has been made accordingly.**

Periods "." missing at the end of lines 119 and 126.

**The corrections have been made accordingly.**

Avoid "HIV+" and "HIV positive" (lines 127, 168, 225) - this formulation is not recommended; use "living with HIV" or "people living with HIV"

**The corrections have been made accordingly.**

Line 144, "and members in the DRIVE-Mind 1", not "and members of the DRIVE-Mind 1"

**The correction has been made accordingly.**

Line 148, 4,500.00 VND? or 4,500,000 VND? In english, we use commas to sepearte integers larger than 1, and a period to indicate decimals. Not sure if there's a 0 missing to make it four and a half million or if the number is four thousand five hundred due to confusing style.

**The reviewer is right, a zero was missing.**

Line 156, avoid "clean" for describing needles - considered stigmatizing since the opposite is "dirty" - please use "sterile" or "non-sterile" when describing injecting equipment.

**The correction has been made accordingly.**

Line 183, an individual can not "use" drug and HIV-relates services - a person can "access" or "engage with" services

**The correction has been made accordingly.**

Line 186, "HIV seroconversion was ascertained by HIV positive rapid tests" - HIV comes up twice here and uses "HIV positive" which is not recommended. I suggest "HIV seroconversion was ascertained by administering the SD bla bla bla testing kit."

**The correction has been made, also taking into account the fact that the editor requests that trademarks be removed from the text.**

Line 224 - suggest reformulating the dates for easier readability - DAY - Month – YEAR

**The correction has been made accordingly.**

Line 237 - a diagnosis cannot be "present" - it can "manifest" or be "detected"

**The correction has been made accordingly.**

Line 244, "visits", not "visit"

**The correction has been made accordingly.**

Line 281, "city" not "City"

**The correction has been made accordingly.**

Line 281, "adherence" not "adhesion"

**The correction has been made accordingly.**

Line 286, no comma after "disorder"

**The correction has been made accordingly.**

Line 189, "an improvement of drug use" - sounds like you are saying that they take better drugs or take drugs more efficiently; I know that's not what the authors meant, but that's what's written! I think the authors meant to say that the PWID "experienced reduced drug-related risks", no?

**The reviewer is absolutely right and the objective was not to say that PWID were using drugs better, but of course that the risks associated with drug use were reduced and also that a reduction in drug use, particularly methamphetamine use, was observed.**

**The sentence has been rephrased as follows: “*Our study suggests that, in addition to a benefit on mental health, this sustained community-based psychiatric intervention is also associated with reduced drug-related risks and substance use (particularly a reduction in methamphetamine use), and improved quality of life in this population*.”**

Similar for Line 296 "benefit on drug use" - which again should be "limited reductions in drug-related risks";

**Thank you again to the reviewer for helping to clarify this point. The intention here was to highlight that the DRIVE-Mind 1 intervention was associated with a limited reduction in substance use. The sentence has been modified accordingly.**

and for Line 299 "get the full benefit of the intervention on drug use" - what is the "full benefit"? What does the mean? do you mean to "maximize the positive impact of the intervention on reducing drug-related risks"?

**What we mean is that extended periods of care are often necessary for the individuals being supported to benefit from the intervention across multiple dimensions. The sentence has been modified as suggested.**

Line 306: "differed for different" is tautological and repetitive, suggest "were different across dimensions" or something like it.

**The correction has been made accordingly.**

Line 310, "had less often a regular salary" should be "very less likely to have a salary"

**The correction has been made accordingly.**

Lines 314-315, "in Vietnam" appears twice in the same sentence

**Thank you again for the reviewer's careful attention.**

Line 323, delete "for most of them" - if you want to say that, I suggest "the majority of CBO members were involved for years..."

**The correction has been made accordingly.**

Line 326-327 is confusing: Suggest edit: "Establishing a network of peers and ensuring official accreditation so that they can effectively deliver mental health services is critical, especially in LMIC where psychiatric resources are most often scarce, given the considerable support then can provide."

**Thanks for the suggestion. The change has been made.**

2- Clarity of statements: There are a few statements that require clarification. The formulation "are/were comparable to a ... population" (lines 30-33, abstract; line 76) is clunky. I think the authors mean to say that the RESULTS were comparable across the two group, but the people are not - the current formulation is inappropriate.

**Thank you again for this comment. We have specified at the end of the sentence "in terms of HIV/HCV exposure", meaning that we are not comparing the populations themselves but rather their HIV/HCV exposure. We hope this clarifies the point.**

Similarly, in lines 233-234, there seems to be a comparison intended, but the comparison isn't explicit - I'm guessing the authors compare the result for the PWID vs control, but that should be explicit.

**The reviewer is right and the sentence has been modified accordingly.**

Line 180, "to assess the severity of the illness" -> was it really the severity of the illness that the authors measured or the severity of the symptoms?

**The CGI scale was designed to allow clinicians to assess the severity of a disorder and the benefit obtained from an intervention, most often pharmacological. Severity is of course assessed through the intensity of symptoms, but also relies on the practitioner's experience, which takes into account various dimensions in the appraisal of this severity. For further reading, the reviewer may refer to the following article, which provides some elements for reflection: *Rabinowitz J, Mehnert A, Eerdekens M. To what extent do the PANSS and CGI-S overlap? J Clin Psychopharmacol. 2006.***

Lines 247-250: This statement is unsupported by evidence from the study - it's a supposition made by the authors that may not be the case. In fact, I think the assertion is confounding the fact that methadone reduces needle sharing which reduces HIV/HCV transmission. So saying that limited enrollment in methadone explains the difference in prevalence between the two groups is spurious. This statement should be reformulated around sharing of injecting equipment rather than injecting or methadone.

**The viral exposure score was constructed as described in the materials and methods section. The combined methadone/injection variable was included, considering that the risk differed considerably during injection practices depending on whether methadone treatment was ongoing or not (data from the literature). The details of the scores are provided in the supplementary data associated with the submission (Table 3). What is reported in the manuscript merely presents the differences shown in the table, without any interpretation on our part.**

Line 290 - The authors mention for the very first tie that the services are co-located - this is really important and should be mentioned and explained well before the discussion section.

**It was specified in the materials and methods section that the intervention took place in the two CBO offices. To remove any ambiguity, it is now clarified in the same section that the psychiatrists intervened directly in the CBO offices.**

Lines 315-316: I don't understand why the authors say that they "could expect an improved access to methadone" since the project wasn't about methadone, didn't offer methadone, or provide referrals to methadone. If it did any of those things, it isn't indicated in the study so the statement feels very out of place and not aligned with the overall study.

**As stated in the initial manuscript (line 155), all study participants benefited from care navigation for HIV when necessary, as well as facilitated access to methadone. This is why we had hoped for a more pronounced benefit in terms of access to methadone.**

Lines 316-319: I don't understand the logic of the statement starting with "If primary-level worker..." - the previous sentence is about methadone (and is confusing), and then the authors switch to "primary-level workers" (which I imagine are peers - if so, say that!) and talk about alcohol which again didn't come up in the article until now. It also feels out of place and out of context.

**Following the reviewer's recommendations, we have removed this sentence.**

Lines 319-320: As the two previous sentence, the statement starting with "Quality of life..." seems out of line with the previous two statements (methadone, peer workers and alcohol) and sounds like an opinion. Why would QoL be "undoubtedly" the last dimension to improve? what's the logic here? where's the evidence to back this up?

**Following the reviewer's recommendations, we have removed this sentence.**

Lines 328-332: I have difficulty that the authors encountered only a single limitation while conducting this study. no issues with laws, policies, law enforcement, safety and security of workers, drop-outs, language, etc? That would be worth a study in and of itself if that was the case!

**The reviewer is right and difficulties did arise during this project, as in any research project. However, several elements that we are unable to describe in detail in this study must be taken into account. First, this project is part of a larger, ongoing initiative known as the DRIVE project, resulting from a research consortium implemented continuously in the city of Haiphong since March 2016 (with more than 35 international publications for the DRIVE-In, DRIVE, DRIVE-C, DRIVE-TB, DRIVE-Mind 1 & 2 projects). Many of the difficulties encountered have therefore largely been overcome since then and did not affect the conduct of our study. The police did intervene at the very beginning of the project in 2016, alerted by the sudden concentration of PWID at the two study sites. However, discussions with provincial authorities, who were supportive of the project, allowed this obstacle to be overcome. Second, we reported the limitation that we considered most significant and most likely to affect the interpretation of the results. Other difficulties are not specific to this intervention (self-reporting bias, etc.). Finally, this study was conducted in 2022–2023, just before legislation in Vietnam became more restrictive. It is clear that we would no longer be able to deploy a project focused on harm reduction for drug users in the current context, or at** **least not in this form.**

3- Data issues: Line 306 - "Data not shown" - why? where is that data?

**These data were not presented in the initial version of the manuscript in order to streamline the presentation of the results. At the request of the reviewer and the editor, they are now included in Supplementary Table 1. The following sentence has been added in the results section: *“Among them, 108 had participated in the DRIVE-Mind 1 study, and 48, who had no psychiatric disorders prior to DRIVE-Mind 2, had been invited to join the control group but were diagnosed with a psychiatric disorder at the initiation of the DM2 project and were therefore referred to the psychiatric cohort (their characteristics are presented in Supplementary Table 1).”***

**Responses to reviewer 2**

**Reviewer #2:** Good work. I have some suggestions and questions below.

In discussion "around 70 percent of the psychiatric cohort had already been engaged in psychiatric care since 2019" If that is the case, why is the intervention effect framed as a 12 month effect rather than as the effect of sustained multi-year care?

**Indeed, an important part of the population was engaged in psychiatric care as part of the previous drive mind 1 study. However, the main objective of the drive-mind 2 study necessitated a control group with an identical monitoring, follow-up and harm reduction support as the group with psychiatric symptoms, and this control group was not implemented at the time of Drive-Mind 1, hence a new study and time frame.**

Rationale for not using matching, inverse probability weighting, or propensity score methods to improve exchangeability between groups before testing noninferiority?

**Methods like propensity score requires large sample and overlap on the main covariates used for matching. A large sample was not possible due to financial constraint (taking into account that this is a prospective cohort) and overlap between groups was less likely to happen since psychiatric status would be the main predictor of intervention. Nevertheless, since the main outcome was viral exposition the two groups were matched according to HIV status.**

Why did you exclude from main analysis 50 control participants who were found to have a psychiatric disorder at M6 and switched into the intervention? In noninferiority settings, post-randomization exclusions or treatment-based exclusions usually bias toward finding noninferiority, not away from it.

**Even though this is not a randomized trial, we applied the same principles for the main analysis: per-protocol is considered the more conservative approach and standard analysis for non-inferiority trials. Therefore, it was decided to include only the participants who received the full course of intervention intended for their initial group. The aim here was to avoid the “dilution” of groups and make sure that any differences between groups regarding the main outcome would appear. This approach allows us to ensure that any similarity in exposure levels is not due to a transfer of participants with the mildest symptoms (i.e. those not detected at the first visit) from the control group to the intervention group.**

How was 0.4 chosen, what real change in HIV or HCV exposure would it correspond to, and what prior evidence supports that this is the largest clinically acceptable loss?

**Since the viral exposition score was created ad hoc, this delta value was not based on literature but established after a discussion among clinicians of the scientific advisory board, during the drafting of the protocol. It corresponds to 2,5% of the total theoretical range of the score (which is 16).** **A preliminary study enabled us to estimate standard deviation of the mean among non-psychiatric participants of the broader DRIVE project, and also helped define this margin of acceptable difference before initiating the study.**

Both groups received peer-supported community services and only one group received psychiatric care, can you disentangle which parts of the observed effect are attributable to psychiatric treatment, peer intensity, or broader social support?

**This is a good point, unfortunately we are not able to quantitatively disentangle these effects since psychiatric care and peer support were strongly linked on visit sites and during linkage to care actions. However, a qualitative approach could allow some granularity on this subject. This could be done in a following study.**

Can you report how many of the 50 controls newly diagnosed at M6 had depression, psychosis, or suicidality, and whether their baseline risk profile resembled the original psychiatric cohort?

**The reviewer will find below 2 tables: the first one with the baseline characteristics of the 266 controls, 156 participants from the psychiatric cohort and 50 from the group who switched from the control group to the psychiatric cohort at M6, and the second one with the psychiatric diagnoses at M6 in the psychiatric cohort and the switch group.**

| **Table 1: M0 Visit comparison between groups** | | | | | | |
| --- | --- | --- | --- | --- | --- | --- |
|  | Control group | Psychiatric cohort | Switch group | p |  | p (comparison psychiatric cohort and switch group only) |
| n | 266 | 156 | 50 |  |  |  |
|  |  |  |  |  |  |  |
| **Age (mean (SD))** | 47.00 (6.84) | 50.78 (8.51) | 49.66 (7.39) | <0.001 |  | 0,404 |
| **Gender : Male (%)** | 253 (94.4) | 141 (90.4) | 48 (96.0) | 0,202 |  | 0,337 |
| **Marital status (%)** |  |  |  | 0,005 |  | 0,34 |
| single | 70 (26.3) | 40 (25.6) | 12 (24.0) |  |  |  |
| legally married | 135 (50.8) | 52 (33.3) | 22 (44.0) |  |  |  |
| living maritally | 6 ( 2.3) | 7 ( 4.5) | 0 ( 0.0) |  |  |  |
| separated | 50 (18.8) | 54 (34.6) | 16 (32.0) |  |  |  |
| widowed | 5 ( 1.9) | 3 ( 1.9) | 0 ( 0.0) |  |  |  |
| **Having a health insurance (%)** | 207 (77.8) | 100 (64.1) | 35 (70.0) | 0,009 |  | 0,553 |
| **Arrested in the last 6 months (%)** | 5 ( 1.9) | 4 ( 2.6) | 1 ( 2.0) | 0,893 |  | 1 |
| **Heroin injection in the last 6 months (%)#** | 115 (43.2) | 68 (43.6) | 22 (44.0) | 0,994 |  | 1 |
| **Heroin injection in the last 30 days (number of days)** | 12.50 [4.00, 30.00] | 17.50 [3.75, 30.00] | 7.50 [1.25, 30.00] | 0,635 |  | 0,252 |
| **Heroin injection (last 30 days)** | 114 (42.9) | 68 (43.6) | 22 (44.0) | 0,982 |  | 1 |
| **Meth use in the last 6 months (%)** | 27 (10.2) | 34 (21.8) | 5 (10.0) | 0,003 |  | 0,1 |
| **Meth use (last 30 days)** | 24 ( 9.0) | 31 (19.9) | 5 (10.0) | 0,005 |  | 0,166 |
| **Meth use ine the last 30 days (number of days, median [IQR])** | 3.50 [2.00, 7.75] | 3.00 [1.00, 5.50] | 5.00 [2.00, 5.00] | 0,63 |  | 0,592 |
| **Regular meth use (>3 times last 30 days)** | 12 ( 4.5) | 13 ( 8.3) | 3 ( 6.0) | 0,276 |  | 0,816 |
| **Alcohol misuse (Audit-C)** | 41 (15.4) | 25 (16.0) | 13 (26.0) | 0,176 |  | 0,17 |
| **Current methadone treatment** | 227 (85.7) | 105 (67.3) | 39 (78.0) | <0.001 |  | 0,209 |
| **Viral exposition score (mean (SD))** | 0.84 (1.02) | 1.03 (1.22) | 0.98 (1.14) | 0,255 |  | 0,813 |

| **Table 2: M6 comparison between groups regarding psychiatric symptoms** | | | |
| --- | --- | --- | --- |
|  |  |  |  |
|  | **Psychiatric cohort** | **Switch group** | **p** |
|  | n=156 | n=50 |  |
| **Current depression (MINI) (%)** |  |  | 0,075 |
| No | 129 (82.7) | 39 (78.0) |  |
| Yes | 19 (12.2) | 11 (22.0) |  |
| NA | 8 ( 5.1) | 0 ( 0.0) |  |
| **Current psychotic disorder (MINI) (%)** |  |  | 0,029 |
| No | 117 (75.0) | 46 (92.0) |  |
| Yes | 31 (19.9) | 4 ( 8.0) |  |
| NA | 8 ( 5.1) | 0 ( 0.0) |  |
| **Suicidal risk (MINI) (%)** |  |  | 0,045 |
| High risk | 8 ( 5.1) | 0 ( 0.0) |  |
| Moderate risk | 6 ( 3.8) | 0 ( 0.0) |  |
| Low risk | 30 (19.2) | 5 (10.0) |  |
| No | 112 (71.8) | 45 (90.0) |  |
| **Other diagnosis (clinical) (%)** |  |  | <0.001 |
| No | 115 (73.7) | 12 (24.0) |  |
| Yes | 33 (21.2) | 38 (76.0) |  |
| NA | 8 ( 5.1) | 0 ( 0.0) |  |
| *Other diagnosis : detail ((%)* |  |  | <0.001 |
| Epilepsia | 2 ( 1.3) | 0 ( 0.0) |  |
| Insomnia | 19 (12.1) | 30 (60.0) |  |
| Anxiety disorders | 11 ( 7.1) | 11 (22.0) |  |
