# Supplementary material for: Benefits of a sustained community-based psychiatric intervention on viral exposure among people who inject drugs: The ANRS Drive-Mind 2 cohort study in Hai Phong, Vietnam
Source: PLOS Ment Health. 2026 Jul 8;3(7):e0000631. doi: 10.1371/journal.pmen.0000631 (PMC13345469; doi:10.1371/journal.pmen.0000631)
Supplement: Peer review history

# Peer review: Decision Letter 0

**DOI:** 10.1371/journal.pmen.0000631.r001

**Authors:** Karli Montague-Cardoso

15 Apr 2026

PMEN-D-25-00585

Benefits of a sustained community-based psychiatric intervention on viral exposure among people who inject drugs: the ANRS Drive-Mind 2 cohort study in Hai Phong, Vietnam

PLOS Mental Health

Dear Dr. Michel,

Thank you for submitting your manuscript to PLOS Mental Health and I am very sorry for the delay in reaching a decision. After careful consideration of the reviewer reports, which we have now received, we feel that your paper has merit but does not fully meet PLOS Mental Health’s publication criteria as it currently stands. Therefore, we invite you to submit a revised version of the manuscript that addresses the points raised during the review process.

Please ensure that all of the comments, which you can find at the end of this email, are fully addressed.

Please submit your revised manuscript by May 29 2026 11:59PM. If you will need more time than this to complete your revisions, please reply to this message or contact the journal office at mentalhealth@plos.org. When you're ready to submit your revision, log on to https://www.editorialmanager.com/pmen/ and select the 'Submissions Needing Revision' folder to locate your manuscript file.

Please include the following items when submitting your revised manuscript:

- A letter that responds to each point raised by the editor and reviewer(s). You should upload this letter as a separate file labeled 'Response to Reviewers'.
- A marked-up copy of your manuscript that highlights changes made to the original version. You should upload this as a separate file labeled 'Revised Manuscript with Track Changes'.
- An unmarked version of your revised paper without tracked changes. You should upload this as a separate file labeled 'Manuscript'.

Guidelines for resubmitting your figure files are available below the reviewer comments at the end of this letter.

As the corresponding author, your ORCID iD is verified in the submission system and will appear in the published article. PLOS supports the use of ORCID, and we encourage all coauthors to register for an ORCID iD and use it as well. Please encourage your coauthors to verify their ORCID iD within the submission system before final acceptance, as unverified ORCID iDs will not appear in the published article. *Only*  the individual author can complete the verification step; PLOS staff *cannot*  verify ORCID iDs on behalf of authors.

We look forward to receiving your revised manuscript.

Kind regards,

Karli Montague-Cardoso

Staff Editor

PLOS Mental Health

**Journal Requirements:**

1. Please include a complete copy of PLOS’ questionnaire on inclusivity in global research in your revised manuscript. Our policy for research in this area aims to improve transparency in the reporting of research performed outside of researchers’ own country or community. The policy applies to researchers who have travelled to a different country to conduct research, research with Indigenous populations or their lands, and research on cultural artefacts. The questionnaire can also be requested at the journal’s discretion for any other submissions, even if these conditions are not met.  Please find more information on the policy and a link to download a blank copy of the questionnaire here: https://journals.plos.org/mentalhealth/s/best-practices-in-research-reporting. Please upload a completed version of your questionnaire as Supporting Information when you resubmit your manuscript.

2. Please amend your detailed Financial Disclosure statement. This is published with the article. It must therefore be completed in full sentences and contain the exact wording you wish to be published.

i. Please clarify all sources of financial support for your study. List the grants, grant numbers, and organizations that funded your study, including funding received from your institution. Please note that suppliers of material support, including research materials, should be recognized in the Acknowledgements section rather than in the Financial Disclosure.

ii. State the initials, alongside each funding source, of each author to receive each grant. For example: "This work was supported by the National Institutes of Health (####### to AM; ###### to CJ) and the National Science Foundation (###### to AM)."

iii. State what role the funders took in the study. If the funders had no role in your study, please state: “The funders had no role in study design, data collection and analysis, decision to publish, or preparation of the manuscript.”

iv. If any authors received a salary from any of your funders, please state which authors and which funders.

3. In the online submission form, you indicated that “The following data will be made available on request addressed to the corresponding author, with publication: de-identified participant data, data dictionary, study protocol, informed consent.”.

All PLOS journals now require all data underlying the findings described in their manuscript to be freely available to other researchers, either

1. In a public repository,

2. Within the manuscript itself, or

3. Uploaded as supplementary information.

This policy applies to all data except where public deposition would breach compliance with the protocol approved by your research ethics board. If your data cannot be made publicly available for ethical or legal reasons (e.g., public availability would compromise patient privacy), please explain your reasons by return email and your exemption request will be escalated to the editor for approval. Your exemption request will be handled independently and will not hold up the peer review process, but will need to be resolved should your manuscript be accepted for publication. One of the Editorial team will then be in touch if there are any issues.

4. Please upload separate figure files in .tif or .eps format. Also, remove the figures from your manuscript file but keep the legends.

For more information about figure files please see our guidelines:

https://journals.plos.org/mentalhealth/s/figures

https://journals.plos.org/mentalhealth/s/figures#loc-file-requirements

5. We noticed that you used “data not shown” in the manuscript. We do not allow these references, as the PLOS data access policy requires that all data be either published with the manuscript or made available in a publicly accessible database. Please amend the supplementary material to include the referenced data or remove the references.

6. We do not publish any copyright or trademark symbols that usually accompany proprietary names, eg (R), (C), or TM  (e.g. next to drug or reagent names). Please remove all instances of trademark/copyright symbols throughout the text, including ‘SD Bioline® HIV’.

7. We have noticed that you have uploaded Supporting Information files, but you have not included a list of legends. Please add a full list of legends for your Supporting Information files after the references list.

If the reviewer comments include a recommendation to cite specific previously published works, please review and evaluate these publications to determine whether they are relevant and should be cited. There is no requirement to cite these works unless the editor has indicated otherwise.

**Additional Editor Comments (if provided):**

[Note: HTML markup is below. Please do not edit.]

Reviewers' comments:

Reviewer's Responses to Questions

**Comments to the Author**

1. Does this manuscript meet PLOS Mental Health’s publication criteria? Is the manuscript technically sound, and do the data support the conclusions? The manuscript must describe methodologically and ethically rigorous research with conclusions that are appropriately drawn based on the data presented.

Reviewer #1: Yes

Reviewer #2: Yes

**********

2. Has the statistical analysis been performed appropriately and rigorously?

Reviewer #1: Yes

Reviewer #2: Yes

**********

3. Have the authors made all data underlying the findings in their manuscript fully available (please refer to the Data Availability Statement at the start of the manuscript PDF file)?

The PLOS Data policy requires authors to make all data underlying the findings described in their manuscript fully available without restriction, with rare exception. The data should be provided as part of the manuscript or its supporting information, or deposited to a public repository. For example, in addition to summary statistics, the data points behind means, medians and variance measures should be available. If there are restrictions on publicly sharing data—e.g. participant privacy or use of data from a third party—those must be specified.

Reviewer #1: No

Reviewer #2: Yes

**********

4. Is the manuscript presented in an intelligible fashion and written in standard English?

PLOS Mental Health does not copyedit accepted manuscripts, so the language in submitted articles must be clear, correct, and unambiguous. Any typographical or grammatical errors should be corrected at revision, so please note any specific errors here.

Reviewer #1: No

Reviewer #2: Yes

**********

5. Review Comments to the Author

Please use the space provided to explain your answers to the questions above. You may also include additional comments for the author, including concerns about dual publication, research ethics, or publication ethics. (Please upload your review as an attachment if it exceeds 20,000 characters)

**Reviewer #1:**  Congratulations on the DRIVE-MIND2 project and an interesting article presenting the findings. That said, there are a few concerns for your consideration:

1- Language: In many instances, the authors use highly clinical language, but in others, the language is very 'community' - I would strongly suggest harmonizing language and terminology so that the article is consistent throughout. For example, the authors use "mental health problem" "disorder" and "condition". "mental health problem" (line 31) and "disorder" (appearing 47 times in the article) are stigmatizing and should be avoided. in most instances, the authors could simply say that psychiatric symptoms were detected. In lines 239-240, the authors say that "all psychiatric disorders significantly decreased" - but a disorder can't be decreased; SYMPTOMS may abate, manifest less frequently, be undetectable or less detectable, etc, but the disorder itself can't be "decreased" in English. Likewise, suggest avoiding "patients" (lines 132, 292) in favor of "study participants" or "PWID".

There are many acronyms that should be spelled out: RDS (line 82), CBO (134), SCDI (135), MMT (155; the authors use methadone, methadone services, etc - use consistent terminology throughout the article), VL (191 - and viral load appears several times out long - either use the acronym consistently or spell out long every time). Some of these acronyms do not appear in he acronyms list a the end.

line 144 has "CBO organizations" which means "community-based organizations organizations" - delete the "organizations" after CBO.

When referring to study timelines, please use "12-month follow-up" (not 12 months follow up). (lines 35, 41, 287)

Line 36 "intervention" not "interventions" (a 12-month ... intervention)

Line 61 "situations" not "situation" (living in precarious social situations)

Study participants can't be "proposed to be recruited" (lines 35, 100, 108, 124, 152, 154) - they were "INVITED to be recruited..."

line 98-99 "PWID PARTICIPATING IN the larger..." not "PWID of the larger..."

Periods "." missing at the end of lines 119 and 126.

Avoid "HIV+" and "HIV positive" (lines 127, 168, 225) - this formulation is not recommended; use "living with HIV" or "people living with HIV"

Line 132, "Patient" should be changed to PWID or study participants (and should be plural).

Line 144, "and members in the DRIVE-Mind 1", not "and members of the DRIVE-Mind 1"

Line 148, 4,500.00 VND? or 4,500,000 VND? In english, we use commas to sepearte integers larger than 1, and a period to indicate decimals. Not sure if there's a 0 missing to make it four and a half million or if the number is four thousand five hundred due to confusing style.

Line 156, avoid "clean" for describing needles - considered stigmatizing since the opposite is "dirty" - please use "sterile" or "non-sterile" when describing injecting equipment.

Line 183, an individual can not "use" drug and HIV-relates services - a person can "access" or "engage with" services

Line 186, "HIV seroconversion was ascertained by HIV positive rapid tests" - HIV comes up twice here and uses "HIV positive" which is not recommended. I suggest "HIV seroconversion was ascertained by administering the SD bla bla bla testing kit."

Line 224 - suggest reformulating the dates for easier readability - DAY - Month - YEAR

Line 237 - a diagnosis cannot be "present" - it can "manifest" or be "detected"

Line 244, "visits", not "visit"

Line 281, "city" not "City"

Line 281, "adherence" not "adhesion"

Line 286, no comma after "disorder"

Line 189, "an improvement of drug use" - sounds like you are saying that they take better drugs or take drugs more efficiently; I know that's not what the authors meant, but that's what's written! I think the authors meant to say that the PWID "experienced reduced drug-related risks", no? Similar for Line 296 "benefit on drug use" - which again should be "limited reductions in drug-related risks"; and for Line 299 "get the full benefit of the intervention on drug use" - what is the "full benefit"? What does the mean? do you mean to "maximize the positive impact of the intervention on reducing drug-related risks"?

Line 306: "differed for different" is tautological and repetitive, suggest "were different across dimensions" or something like it.

Line 310, "had less often a regular salary" should be "very less likely to have a salary"

Lines 314-315, "in Vietnam" appears twice in the same sentence

Line 323, delete "for most of them" - if you want to say that, I suggest "the majority of CBO members were involved for years..."

Line 326-327 is confusing: Suggest edit: "Establishing a network of peers and ensuring official accreditation so that they can effectively deliver mental health services is critical, especially in LMIC where psychiatric resources are most often scarce, given the considerable support then can provide."

2- Clarity of statements: There are a few statements that require clarification. The formulation "are/were comparable to a ... population" (lines 30-33, abstract; line 76) is clunky. I think the authors mean to say that the RESULTS were comparable across the two group, but the people are not - the current formulation is inappropriate. Similarly, in lines 233-234, there seems to be a comparison intended, but the comparison isn't explicit - I'm guessing the authors compare the result for the PWID vs control, but that should be explicit.

Line 180, "to assess the severity of the illness" -> was it really the severity of the illness that the authors measured or the severity of the symptoms?

Lines 247-250: This statement is unsupported by evidence from the study - it's a supposition made by the authors that may not be the case. In fact, I think the assertion is confounding the fact that methadone reduces needle sharing which reduces HIV/HCV transmission. So saying that limited enrollment in methadone explains the difference in prevalence between the two groups is spurious. This statement should be reformulated around sharing of injecting equipment rather than injecting or methadone.

Line 290 - The authors mention for the very first tie that the services are co-located - this is really important and should be mentioned and explained well before the discussion section.

Lines 315-316: I don't understand why the authors say that they "could expect an improved access to methadone" since the project wasn't about methadone, didn't offer methadone, or provide referrals to methadone. If it did any of those things, it isn't indicated in the study so the statement feels very out of place and not aligned with the overall study.

Lines 316-319: I don't understand the logic of the statement starting with "If primary-level worker..." - the previous sentence is about methadone (and is confusing), and then the authors switch to "primary-level workers" (which I imagine are peers - if so, say that!) and talk about alcohol which again didn't come up in the article until now. It also feels out of place and out of context.

Lines 319-320: As the two previous sentence, the statement starting with "Quality of life..." seems out of line with the previous two statements (methadone, peer workers and alcohol) and sounds like an opinion. Why would QoL be "undoubtedly" the last dimension to improve? what's the logic here? where's the evidence to back this up?

Lines 328-332: I have difficulty that the authors encountered only a single limitation while conducting this study. no issues with laws, policies, law enforcement, safety and security of workers, drop-outs, language, etc? That would be worth a study in and of itself if that was the case!

3- Data issues: Line 306 - "Data not shown" - why? where is that data?

**Reviewer #2:**  Good work. I have some suggestions and questions below.

In discussion "around 70 percent of the psychiatric cohort had already been engaged in psychiatric care since 2019" If that is the case, why is the intervention effect framed as a 12 month effect rather than as the effect of sustained multi-year care?

Rationale for not using matching, inverse probability weighting, or propensity score methods to improve exchangeability between groups before testing noninferiority?

Why did you exclude from main analysis 50 control participants who were found to have a psychiatric disorder at M6 and switched into the intervention? In noninferiority settings, post-randomization exclusions or treatment-based exclusions usually bias toward finding noninferiority, not away from it.

How was 0.4 chosen, what real change in HIV or HCV exposure would it correspond to, and what prior evidence supports that this is the largest clinically acceptable loss?

Both groups received peer-supported community services and only one group received psychiatric care, can you disentangle which parts of the observed effect are attributable to psychiatric treatment, peer intensity, or broader social support?

Can you report how many of the 50 controls newly diagnosed at M6 had depression, psychosis, or suicidality, and whether their baseline risk profile resembled the original psychiatric cohort?

Thanks and regards

**********

6. PLOS authors have the option to publish the peer review history of their article (what does this mean?). If published, this will include your full peer review and any attached files.

**Do you want your identity to be public for this peer review?**  If you choose “no”, your identity will remain anonymous but your review may still be made public.

For information about this choice, including consent withdrawal, please see our Privacy Policy.

Reviewer #1: **Yes:**  Pascal Tanguay

Reviewer #2: **Yes:**  Dr Parul Parul

**********

[NOTE: If reviewer comments were submitted as an attachment file, they will be attached to this email and accessible via the submission site. Please log into your account, locate the manuscript record, and check for the action link "View Attachments". If this link does not appear, there are no attachment files.]

**Figure Resubmissions:**

While revising your submission, we strongly recommend that you use PLOS’s NAAS tool (https://ngplosjournals.pagemajik.ai/artanalysis) to test your figure files. NAAS can convert your figure files to the TIFF file type and meet basic requirements (such as print size, resolution), or provide you with a report on issues that do not meet our requirements and that NAAS cannot fix.

After uploading your figures to PLOS’s NAAS tool - https://ngplosjournals.pagemajik.ai/artanalysis, NAAS will process the files provided and display the results in the "Uploaded Files" section of the page as the processing is complete. If the uploaded figures meet our requirements (or NAAS is able to fix the files to meet our requirements), the figure will be marked as "fixed" above. If NAAS is unable to fix the files, a red "failed" label will appear above. When NAAS has confirmed that the figure files meet our requirements, please download the file via the download option, and include these NAAS processed figure files when submitting your revised manuscript.

# Author comment: Author response to Decision Letter 1

**DOI:** 10.1371/journal.pmen.0000631.r002

3 May 2026

Attachment Submitted filename: Response to reviewers.docx

# Peer review: Decision Letter 1

**DOI:** 10.1371/journal.pmen.0000631.r003

**Authors:** Karli Montague-Cardoso, Karli Montague-Cardoso

15 May 2026

Benefits of a sustained community-based psychiatric intervention on viral exposure among people who inject drugs: the ANRS Drive-Mind 2 cohort study in Hai Phong, Vietnam

PMEN-D-25-00585R1

Dear Dr Michel,

We are pleased to inform you that your manuscript 'Benefits of a sustained community-based psychiatric intervention on viral exposure among people who inject drugs: the ANRS Drive-Mind 2 cohort study in Hai Phong, Vietnam' has been provisionally accepted for publication in PLOS Mental Health.

Before your manuscript can be formally accepted you will need to complete some formatting changes, which you will receive in a follow up email. A member of our team will be in touch with a set of requests.

Please note that your manuscript will not be scheduled for publication until you have made the required changes, so a swift response is appreciated.

IMPORTANT: The editorial review process is now complete. PLOS will only permit corrections to spelling, formatting or significant scientific errors from this point onwards. Requests for major changes, or any which affect the scientific understanding of your work, will cause delays to the publication date of your manuscript.

If your institution or institutions have a press office, please notify them about your upcoming paper to help maximize its impact. If they'll be preparing press materials, please inform our press team as soon as possible -- no later than 48 hours after receiving the formal acceptance. Your manuscript will remain under strict press embargo until 2 pm Eastern Time on the date of publication. For more information, please contact mentalhealth@plos.org.

Thank you again for supporting Open Access publishing; we are looking forward to publishing your work in PLOS Mental Health.

Best regards,

Karli Montague-Cardoso

Staff Editor

PLOS Mental Health

***********************************************************

Reviewer Comments (if any, and for reference):

Reviewer's Responses to Questions

**Comments to the Author**

1. If the authors have adequately addressed your comments raised in a previous round of review and you feel that this manuscript is now acceptable for publication, you may indicate that here to bypass the “Comments to the Author” section, enter your conflict of interest statement in the “Confidential to Editor” section, and submit your "Accept" recommendation.

Reviewer #1: All comments have been addressed

**********

2. Does this manuscript meet PLOS Mental Health’s publication criteria? Is the manuscript technically sound, and do the data support the conclusions? The manuscript must describe methodologically and ethically rigorous research with conclusions that are appropriately drawn based on the data presented.

Reviewer #1: Yes

**********

3. Has the statistical analysis been performed appropriately and rigorously?

Reviewer #1: I don't know

**********

4. Have the authors made all data underlying the findings in their manuscript fully available (please refer to the Data Availability Statement at the start of the manuscript PDF file)?

The PLOS Data policy requires authors to make all data underlying the findings described in their manuscript fully available without restriction, with rare exception. The data should be provided as part of the manuscript or its supporting information, or deposited to a public repository. For example, in addition to summary statistics, the data points behind means, medians and variance measures should be available. If there are restrictions on publicly sharing data—e.g. participant privacy or use of data from a third party—those must be specified.

Reviewer #1: Yes

**********

5. Is the manuscript presented in an intelligible fashion and written in standard English?

PLOS Mental Health does not copyedit accepted manuscripts, so the language in submitted articles must be clear, correct, and unambiguous. Any typographical or grammatical errors should be corrected at revision, so please note any specific errors here.

Reviewer #1: Yes

**********

6. Review Comments to the Author

Please use the space provided to explain your answers to the questions above. You may also include additional comments for the author, including concerns about dual publication, research ethics, or publication ethics. (Please upload your review as an attachment if it exceeds 20,000 characters)

Reviewer #1: All my comments and suggestions adequately addressed

**********

7. PLOS authors have the option to publish the peer review history of their article (what does this mean?). If published, this will include your full peer review and any attached files.

**Do you want your identity to be public for this peer review?**  If you choose “no”, your identity will remain anonymous but your review may still be made public.

For information about this choice, including consent withdrawal, please see our Privacy Policy.

Reviewer #1: **Yes:**  Pascal Tanguay

**********
